# Old Bottle, New Wine: Diplopia Sans Proptosis as the Sole Presentation of Thyroid Orbitopathy

**DOI:** 10.7759/cureus.15850

**Published:** 2021-06-23

**Authors:** Divyani Garg, Ankit Gupta, Rajinder K Dhamija

**Affiliations:** 1 Neurology, Lady Hardinge Medical College, Delhi, IND; 2 Neurology, Lady Hardinge Medical College, New Delhi, IND

**Keywords:** thyroid orbitopathy, diplopia, hypothyroidism, graves’ disease, proptosis

## Abstract

Thyroid orbitopathy holds a dominant place in the list of causes for external ophthalmoplegia and is commonly accompanied by eye surface signs such as congestion, lacrimation, and proptosis. An elderly male presented to us with painless diplopia in the absence of proptosis, conjunctival congestion, tearing or irritative symptoms. He had bilateral complete external ophthalmoplegia. He was evaluated to have hypothyroidism with elevated anti-thyroid peroxidase antibodies. MRI revealed bulky extraocular muscles in a pattern of sparing of tendinous insertions, highly suggestive of thyroid orbitopathy. He was managed with IV steroid pulse and thyroxine supplementation with minimal improvement.

Through this case report, we highlight an unusual presentation of thyroid eye disease in the form of isolated diplopia, in the absence of other usual eye signs. We also emphasize the characteristic typical neuroimaging signs, such as the ‘Coca Cola bottle’ sign, which strongly augments the diagnosis in an atypical setting.

## Introduction

Thyroid orbitopathy is a rare condition, with a reported incidence of 2.9 to 16 per million population per year [1]. Most patients develop this condition in the setting of Graves’ disease. Some 10%-15% of cases are present in association with current or past history of hypothyroidism [2]. Usual ophthalmologic complaints include eye surface symptoms such as redness or irritation, along with exophthalmos, retro-orbital pain, and restrictive ophthalmoplegia. Diplopia as the sole manifestation of thyroid orbitopathy is relatively uncommon and must trigger a search for alternate causes [3].

## Case presentation

**Figure 1:**
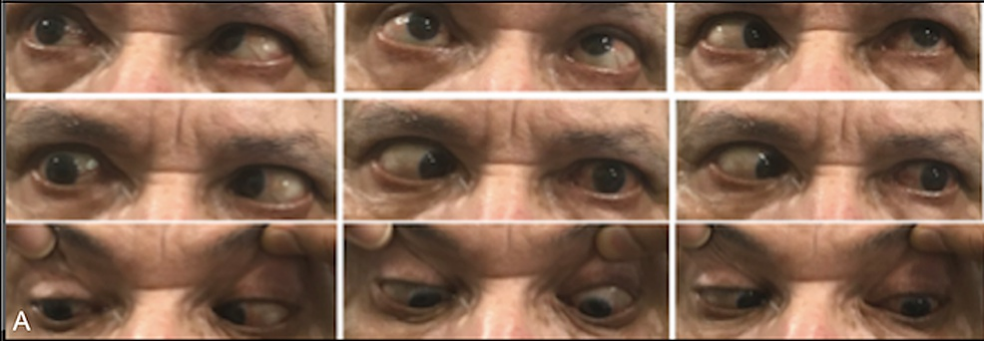
Examination in the nine cardinal positions of gaze reveals bilateral ophthalmoplegia in all directions.

A 65-year-old male presented with progressive binocular horizontal diplopia along with an inability to move his eyeballs in any direction for seven months which worsened on looking towards the right without a history of ptosis, fatigability, or diurnal fluctuation of symptoms. He had no eyeball prominence, congestion, irritation, lacrimation, blurring of vision, or pain on eyeball movement. He was hypertensive with stage 3 chronic kidney disease and had generalized vitiligo, with no relevant family history. Examination revealed right eye esotropia with bilateral complete gaze restriction, without ptosis, proptosis, conjunctival congestion, or lacrimation (Figure 1).

Fundus examination showed bilateral grade 2 hypertensive retinopathy. Visual acuity, intraocular pressure, and pupillary examination were normal. Clinical tests of fatigability and ice pack test were negative. The remaining neurological and systemic examination was normal.

**Figure 2:**
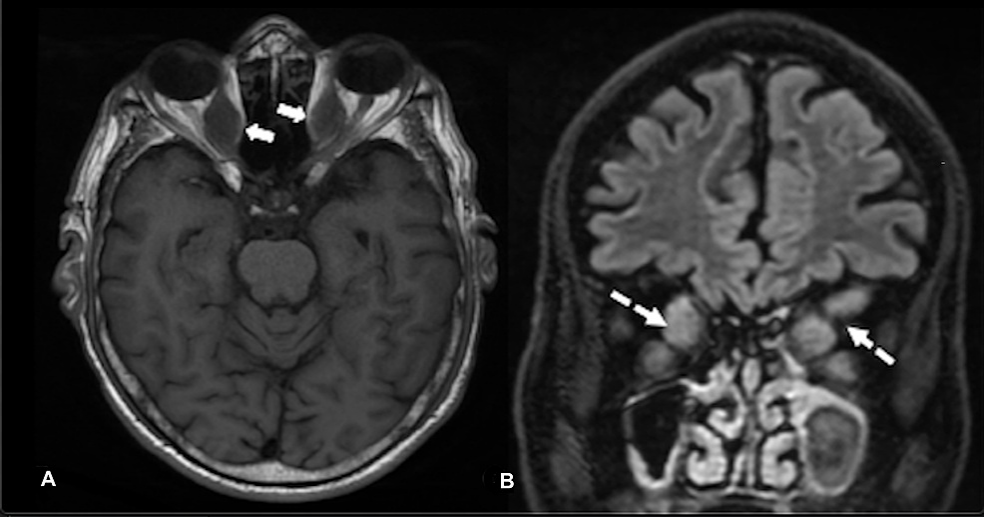
MRI brain demonstrating the 'Coca Cola Bottle' sign. A. MRI brain (axial section) T1 sequence shows symmetrically enlarged medial rectus muscle bellies (closed arrows) with sparing of the tendinous insertions, suggestive of the ‘Coca Cola Bottle’ sign. B. MRI brain (coronal section) T2/FLAIR sequence shows enlarged medial, superior, and inferior rectus muscle bellies (dashes arrows).

Routine investigations, including complete blood count and hepatic function tests, were normal. Renal function tests revealed creatinine of 2.5 mg/dL, urea 40 mg/dL, sodium 138 meq/L, and potassium 4.1 meq/L. Thyroid function tests showed thyroid stimulating hormone (TSH)-85.26 mIU/L and fT4-0.56 ng/dL (normal: 0.7-1.9). Anti-thyroid peroxidase (TPO) antibodies were highly elevated (>1300 IU/mL, normal <35 IU/mL). Blood sugar levels and antinuclear antibody enzyme immunoassay (EIA) were negative. Serum angiotensin converting enzyme (ACE) levels were within normal limits. MRI brain revealed gross enlargement of all extraocular muscles symmetrically sparing the tendinous insertions except the lateral rectus muscles on both sides, impinging on the right optic nerve (Figure 2).

This was consistent with the ‘Coca-Cola Bottle’ sign described in thyroid orbitopathy classically [4]. The cerebrospinal fluid (CSF) examination was normal. He was initiated on thyroxine supplementation and IV methylprednisolone pulse 500 mg per week for 12 weeks with which he showed minimal improvement.

## Discussion

Thyroid orbitopathy is an autoimmune process due to shared autoantigens between thyroid tissue and the orbit. Lymphocytic infiltration into the extraocular muscles and retro-orbital tissue leads to cytokine activation and inflammation of the extraocular muscles [3]. Orbital fibroblasts secrete excess glycosaminoglycan leading to bulking of extraocular muscles, retro-orbital fat, and peri-orbital tissue. Smoking increases the risk of development of thyroid orbitopathy by seven to eight times [5].

Unusual features in our case included the occurrence of hypothyroidism and the absence of other ocular findings. The majority of patients with thyroid orbitopathy have Graves’ disease, although it may occur in thyroid cancers, autoimmune thyroiditis, and without thyroid disease [6]. Some 10%-15% of patients may have hypothyroidism, like our patient. In 10%, thyroid ophthalmopathy may precede thyroid disease [2].

Although thyroid orbitopathy is 2.5-6 times more common in females, the more severe affliction occurs in males, and after 50 years of age [7]. Rundle’s curve describes the natural course of thyroid eye disease, according to which eye disease worsens to a peak in a few months after which it improves spontaneously [2]. This may continue for a few years after which the disease enters the ‘burnt-out’ phase when no further changes occur. The first phase with progressive worsening defines the ‘active’ phase during which medical treatment is believed to be effective. ‘Inactive’ disease suggests that medical therapy is ineffective, and the patient may respond only to surgical treatment. We believe that our patient was in the active phase as he had progressive worsening of diplopia and hence, we treated him with steroids along with levothyroxine supplementation. Smoking cessation was also advised since it exacerbates thyroid eye disease and also reduces treatment efficacy [8].

Treatment is divided into (a) management of vision-threatening complications (exposure keratopathy or optic nerve compression); (b) avoidance of factors that exacerbate thyroid eye disease (smoking cessation/correcting thyroid dysfunction); and (c) specialist center referral (when needed) [3].

Steroids are the most important treatment modality and lead to symptomatic improvement in one to two weeks. Not only are they anti-inflammatory but also suppress the secretion of glycosaminoglycans from orbital fibroblasts [3]. A pulse therapy of 500-1000 mg for three to five days followed by oral steroids is preferred over daily oral treatment alone. Weekly pulse steroid cycles were found to be more effective and had lesser side effects than daily oral steroid treatment [8]. Immunosuppressants like rituximab, ciclosporin, azathioprine, and methotrexate are also used, although their efficacy is still uncertain. Euthyroidism leads to symptomatic improvement in a few months and is essential, as thyroid dysfunction has adverse outcomes [8]. Radio-active iodine is best avoided in the active phase of disease due to a small, reported risk of exacerbation [9].

Surgery has a role in all disease phases. In the active phase, urgent orbital decompression may be considered for patients with optic neuropathy not responding to/tolerating steroids, or corneal ulceration with associated exophthalmos. Rehabilitative reconstructive surgery for cosmetic purposes may be offered in the inactive phase [3, 8].

Visual acuity must be paid careful attention to. Often, the pressure within the globe is attenuated by spontaneous exophthalmos of the eye. The absence of exophthalmos in patients with thyroid eye disease has been uncommonly reported. In these cases, the orbital pressure decompresses by compressing on the globe, causing a rise in intraocular pressure. However, our patient had normal intraocular pressures, probably related to ongoing intraorbital compensation.

The MRI features of muscle bulking are typical with sparing of tendinous insertions. Other differentials include orbital pseudotumor, lymphoma, metastasis, sarcoidosis, and arteriovenous malformation. However, these are usually unilateral and asymmetrical (if bilateral) and tend to involve the tendinous ring [10].

## Conclusions

Thyroid eye disease holds a dominant place in the list of causes for external ophthalmoplegia. The diagnosis is strongly augmented in the presence of typical neuroimaging signs such as the ‘Coca Cola bottle’ sign. Treatment requires correction of the dysthyroid state and steroid therapy, the decision for which may be based on ongoing disease activity.

## References

[1] Bartley GB, Fatourechi V, Kadrmas EF, Jacobsen SJ, Ilstrup DM, Garrity JA, Gorman CA (1995). The incidence of Graves' ophthalmopathy in Olmsted County, Minnesota. Am J Ophthalmol.

[2] Wiersinga WM (2007). Management of Graves' ophthalmopathy. Nat Clin Pract Endocrinol Metab.

[3] Perros P, Neoh C, Dickinson J (2009). Thyroid eye disease. BMJ.

[4] Gonçalves AC, Gebrim EM, Monteiro ML (2012). Imaging studies for diagnosing Graves' orbitopathy and dysthyroid optic neuropathy. Clinics (Sao Paulo).

[5] Phillips ME, Marzban MM, Kathuria SS (2010). Treatment of thyroid eye disease. Curr Treat Options Neurol.

[6] Orgiazzi J (2008). Pathogenesis. Graves’ Orbitopathy. A Multidisciplinary Approach.

[7] Şahlı E, Gündüz K (2017). Thyroid-associated ophthalmopathy. Turk J Ophthalmol.

[8] Verity DH, Rose GE (2013). Acute thyroid eye disease (TED): principles of medical and surgical management. Eye (Lond).

[9] Bartalena L, Tanda ML, Piantanida E, Lai A (2005). Glucocorticoids and outcome of radioactive iodine therapy for Graves' hyperthyroidism. Eur J Endocrinol.

[10] Parmar H, Ibrahim M (2008). Extrathyroidal manifestations of thyroid disease: thyroid ophthalmopathy. Neuroimaging Clin N Am.

